# The economic impact of a physician-introduced web portal on lower back pain: results from a cluster-randomized trial

**DOI:** 10.1186/s12962-026-00796-z

**Published:** 2026-07-07

**Authors:** Klaus Kaier, Christian Schlett, Piet van der Keylen, Renate Lange, Andrea C. Schöpf-Lazzarino, Martin Boeker, Mirjam Körner, Andy Maun, Sebastian Voigt-Radloff, Erik Farin-Glattacker

**Affiliations:** 1 Institute of Medical Biometry and Statistics, Medical Center – University of Freiburg, Faculty of Medicine, University of Freiburg, Freiburg, Germany; 2 Section of Health Care Research and Rehabilitation Research, Medical Center – University of Freiburg, Faculty of Medicine, Freiburg, Germany; 3 Institute of General Practice, Friedrich-Alexander University Erlangen-Nürnberg, University Hospital Erlangen, Erlangen, Germany; 4 Professorship of Medicine and Health Sciences, Lutheran Univeristy of Applied Sciences Nürnberg, Nürnberg, Germany; 5 BKK Landesverband Bayern, Munich, Germany; 6 Careum School of Health, Zurich, Switzerland; 7 Institute for Artificial Intelligence and Informatics in Medicine, Technical University of Munich, Munich, Germany; 8 Institute of Medical Psychology and Medical Sociology, Faculty of Medicine, University of Freiburg, Freiburg, Germany; 9 Department of Health Professions, Competence Centre Interprofessionalism, Bern University of Applied Sciences, Bern, Switzerland; 10 Institute of General Practice / Family Medicine, Medical Center – University of Freiburg, Faculty of Medicine, Freiburg, Germany; 11 Institute for Evidence in Medicine, Faculty of Medicine and Medical Center, University of Freiburg, Freiburg, Germany

## Abstract

**Background:**

Low back pain (LBP) is a prevalent condition that imposes a significant economic burden on healthcare systems. Digital health interventions, such as the web-based tala-med platform, offer an evidence-based approach to patient education and shared decision-making. This study evaluates the financial and cost-effectiveness implications of integrating tala-med into primary care compared to routine care.

**Methods:**

This multi-center, cluster-randomized trial assigned primary care practices (PCPs) in Germany to either an intervention group (IG) using tala-med or a control group (CG) providing routine care. Patients aged ≥ 18 with LBP were recruited and health economic evaluation included a claims-data-based cost analysis of back pain-related healthcare utilization for a three month follow-up. Cost-effectiveness was assessed by comparing additional costs per unit increase in self-reported knowledge and perceived informedness. LBP knowledge ranged from 1 (very low) to 5 (very high) and LBP informedness ranged from 1 (not at all) to 5 (very good). Sensitivity analyses included also non-back-pain related healthcare utilization and restricted the analysis to a sub-cohort of currently employed patients.

**Results:**

Data from 183 intervention and 133 control patients were analysed. The intervention group demonstrated fewer general practitioner (risk-adjusted incidence rate ratio (IRR): 0.86, *p* = 0.284) and specialist contacts (IRR 0.70, *p* = 0.009), but significantly more back pain-related sick leave (IRR 8.73, *p* = 0.001). The adjusted increase in back pain-related costs in the IG was €822.22 (*p* = 0.005). Sensitivity analyses showed minimal impact on total healthcare costs (€145.88, *p* = 0.819). Cost-effectiveness analysis indicated additional costs of €3328.54 per unit increase in self-reported knowledge and €1611.21 per unit increase in perceived informedness.

**Discussion:**

While tala-med use was associated with increased back pain-related costs, total healthcare expenditures remained stable. The reasons for the discrepancy remain unclear but may include increased awareness of LBP or extended recovery periods facilitated by sick leave. Further research is needed to validate these findings and explore the long-term benefits of digital health interventions in LBP management.

**Supplementary Information:**

The online version contains supplementary material available at 10.1186/s12962-026-00796-z.

## Introduction

Lower back pain (LBP) is a leading cause of primary care visits and significantly impacts healthcare and quality of life [1–3]. In Germany, over 70% of adults experience LBP annually, making it one of the most searched health topics online [4, 5]. Since COVID-19, both patients and primary care physicians (PCPs) increasingly rely on the internet for health information, but reliable, comprehensible resources remain scarce [6–8].

Despite national and international guidelines, LBP treatment options vary widely, leading to confusion among patients and challenges for PCPs in accessing up-to-date, evidence-based information [9–11]. Digital health interventions have shown small but meaningful benefits for LBP patients, yet gaps remain in patient knowledge [12–16].

To address this, the GAP-Project developed tala-med, a web portal providing evidence-based, accessible LBP information for both patients and PCPs [17]. Tala-med aims to improve patient knowledge, self-management, shared decision-making, and patient-physician communication [17].

This study evaluates the financial and cost-effectiveness implications of integrating tala-med into primary care compared to routine care.

## Methods

This multi-center, cluster-randomized trial compared tala-med use to routine care in LBP patients. PCP practices were randomized (2:1) into intervention (IG) or control (CG) groups, stratified by PCP count per practice. The 2:1 allocation ratio was prespecified in the trial protocol [17] to maximise the number of patients and physicians exposed to the portal — supporting the assessment of portal usage and the process evaluation — while retaining a control group adequate for the primary comparison.

PCPs in Germany recruited LBP patients that were aged ≥ 18 and insured in one of the many company health insurance funds in Bavaria, Germany. More details on the study design, outcomes, recruitment and questionnaires are described the main article of the study [18]. The state association of company health insurance funds in Bavaria asked the responsible company health insurance about days of sick leave and utilisation of the healthcare system for a three month follow-up period. In the health-economic evaluation, all patients were included for whom the company health insurance funds (BKK) were able to provide routine claims data. This applied to 183 of 190 included patients in the intervention group and to 133 of 139 included patients in the control group. Consequently, the health-economic analysis population differs slightly from the effectiveness-analysis population reported in the main trial publication [18], because for a few patients not both types of information — evaluable questionnaire data and routine claims data — were available. PCPs received financial incentives for recruitment and tala-med use, while patients received a book voucher for follow-up questionnaire completion. Tala-med, a German-language web portal, provides evidence-based LBP information based on national and international guidelines. Developed by a multidisciplinary team, it includes tailored versions for PCPs and patients. PCPs used tala-med during consultations and encouraged patients’ use at home. The patient version offers educational materials, multimedia content, exercise videos, and shared decision-making resources. PCPs and patients accessed their respective versions via individual log-ins during the trial. Tala-med became publicly available after the study (https://ruecken.tala-med.info/) and has remained freely available since then.

We conducted a claims-data-based cost analysis over the three-month follow-up period. Direct medical utilisation was analysed from the perspective of the statutory health insurance, while sickness absence was additionally valued as a productivity-cost component using the human capital method. The tala-med portal was used within an already-reimbursed primary care consultation and did not constitute a separately billable service, medical device, prescribed therapy, or reimbursed digital health application. Trial-related payments to physicians, including recruitment incentives and the one-off payment for the first portal-supported consultation, were research and implementation incentives rather than routine-care costs and were therefore not included in the claims-data-based cost analysis. For this purpose, only secondary outcomes, i.e. the costs of back pain-related utilisation of the healthcare system and the costs of incapacity for work, were considered. The following cost indicators were used at individual patient level for the three-month observation period following the declaration of consent: number of general practitioner contacts, number of specialist contacts, number of hospital days, total hospital costs, number of days of sick leave. Standardised unit costs were used to price the use of outpatient services [19]. The human capital method was used to price the days of sick leave. Indirect costs of €332 were used for this [20], which are made up of the loss of production (€123) and the loss of gross value added (€209). All prices and costs refer to the base year 2019, representing the midpoint of the 2018–2020 recruitment window. The identification of back pain-related costs for the healthcare system was based on predefined main diagnoses and procedures (see supplemental Table 1). This set of diagnoses and procedures was prespecified in the study protocol and agreed within an interdisciplinary expert panel during the design of the study. Recurrent and depressive episodes (F32, F33) and non-specific pain (R52) were included because they frequently co-occur with, and form part of the care pathway for, chronic and somatising back pain.

The primary analysis of the financial effect of the intervention took into account the back pain-related inpatient and outpatient costs for the healthcare system and the costs for back pain-related days of incapacity for work, which were assessed using the human capital method, as the actual costs of sick days were not available via the routine data. In sensitivity analyses, the approach was repeated for (1) total, i.e. not only back pain-related, medical service utilisation and (2) for the sub-cohort of currently employed patients. In order to compensate for remaining differences in patient characteristics between the intervention and control groups, a propensity score was determined from the patient characteristics of age and gender, for which adjustments were made in all regression analyses [21]. Because randomisation was performed at the cluster (practice) level, balance in patient characteristics is primarily achieved through the cluster randomisation; the propensity score for age and sex was included to account for the most relevant residual imbalance in patient characteristics. As a non-prespecified sensitivity analysis, we additionally adjusted the cost analyses for prior healthcare utilisation, operationalised as the total cost of healthcare utilisation in the three months prior to study inclusion. This variable was selected because it captures the most substantial part of the patient-level baseline differences in care-seeking that are not already addressed by the cluster randomisation. The results of this additional analysis are shown in Figure S1.

For the analysis of count data, poisson regression models were used with exponentiated coefficients interpreted as incidence rate ratios. Two-part regression models with a logistic and a generalised linear model with log-link and gamma distribution were selected for the analysis of cost data in order to take into account the right skewness that is common in cost data [22]. The two-part model specification was prespecified on the basis of established practice and prior experience with right-skewed cost data; no formal goodness-of-fit testing or comparison with alternative distributional assumptions was performed. In addition, cluster randomisation was taken into account by specifying cluster-robust standard errors at the level of the recruiting physician/practice cluster in all regression analyses.

In a second step, the results of the claims-data-based cost analysis were compared with the primary outcomes, i.e. ‘self-reported knowledge’ and ‘perceived informedness’, as part of an exploratory cost-effectiveness analysis. The questionnaires for measuring ‘self-reported knowledge’ and ‘perceived informedness’ were developed as part of this study, and were described in detail in the recently published main article of the study [18]. The aim was to determine two cost-effectiveness ratios: Additional costs per unit of ‘perceived informedness’ and additional costs per unit of ‘self-reported knowledge’ on the patient side. ‘self-reported knowledge’ ranged from 1 (very low) to 5 (very high) and ‘perceived informedness’ranged from 1 (not at all) to 5 (very good). Confidence intervals for the cost-effectiveness comparisons were determined using Fieller’s theorem [23–25]. Separately, the 95% confidence ellipses surrounding the mean estimates on the cost-effectiveness planes were derived from 1,000 bootstrap replications [26–28]; the same replications were used to construct the cost-effectiveness acceptability curves [29], which indicate the probability that the intervention is considered cost-effective for different values of the willingness to pay for a one-fold improvement of the patients ‘self-reported knowledge’ and ‘perceived informedness’. The trial sample size was determined on the basis of the primary clinical outcomes reported in the main publication [18]. No separate power or sample size calculation was performed for the health economic outcomes, which were analysed as secondary, exploratory endpoints.

## Results

Data from 183 patients in the intervention group and 133 patients in the control group were provided for the analysis. 52% of the participants in the intervention group were female (control group: 62%), on average 46 years old (control group: 52 years) and for the most part employed (81% vs. 68%; Table 1). Patients were nested within the recruiting physicians/practices. In the health-economic complete-case analysis, the 133 control-group patients were nested within 12 cluster IDs and the 183 intervention-group patients within 34 cluster IDs.

**Table 1:** Baseline characteristics

Female (*N*, %)		Control group (*N* = 133)			Intervention group (*N* = 183)	
		83	62.41%		95	51.91%
Age (mean, SD)		52.1	14.22		45.68	12.97
currently employed (N, %)		90	67.67%		149	81.42%
year of study enrolment 2018		26	19.55%		68	37.16%
2019		83	62.41%		90	49.18%
2020		24	18.05%		25	13.66%

### Main analysis: Back pain-related healthcare service utilization in the overall cohort

**Fig. 1:**
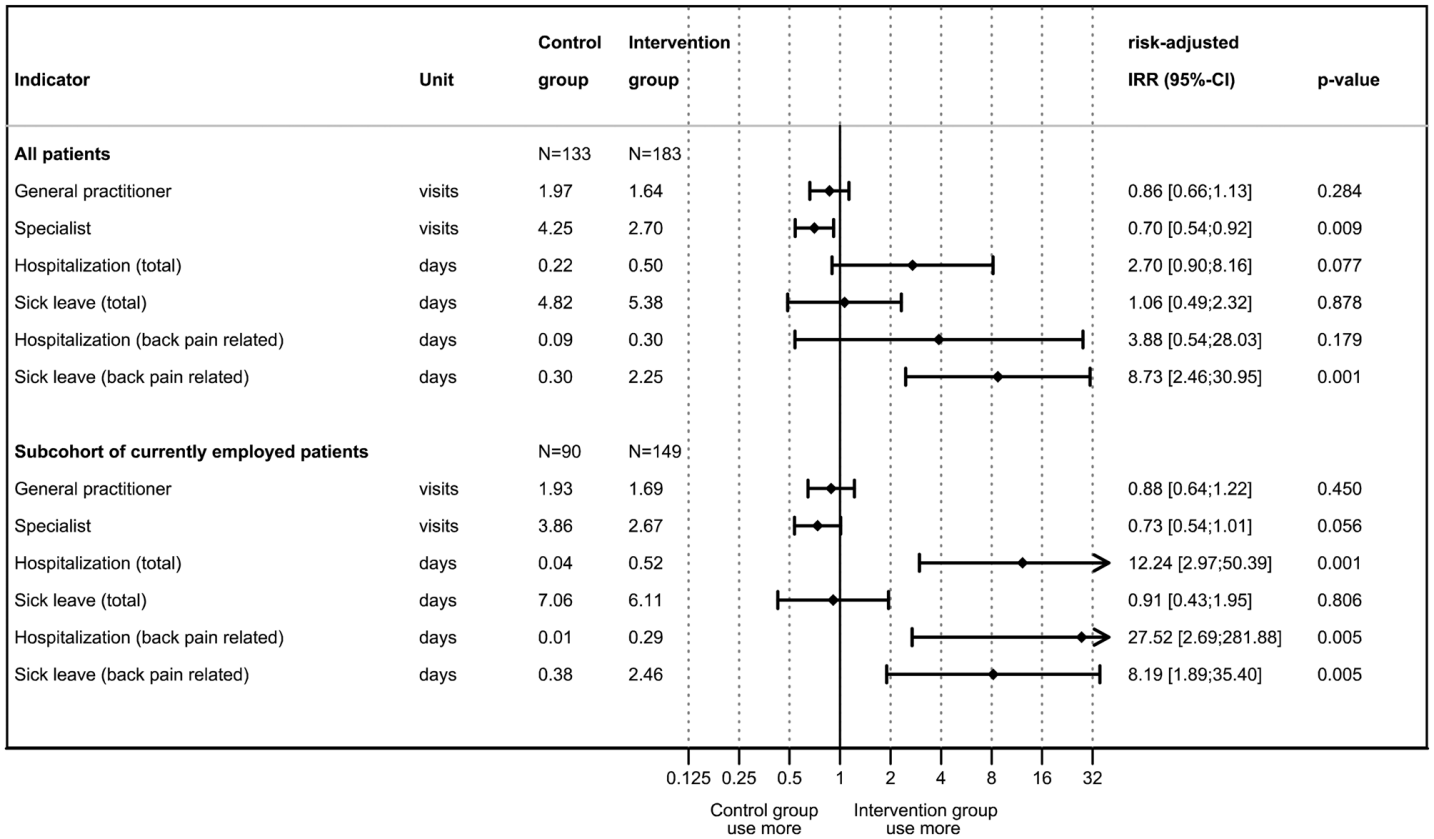
Adjusted differences in medical service utilisation

Figure 1 displays a heterogeneous picture regarding the financial effect of the intervention from the perspective of the health insurance companies. In the 3-month period, the intervention group demonstrated fewer general practitioner (risk-adjusted incidence rate ratio (IRR): 0.86, *p* = 0.284) and specialist contacts (IRR 0.70, *p* = 0.009) but significantly more back pain-related sick leave (IRR 8.73, *p* = 0.001). Expressed in absolute terms, back pain-related sick leave averaged 2.25 days per patient in the intervention group versus 0.30 days in the control group over the three-month period, whereas total (all-cause) sick leave was similar between groups (intervention 5.38 vs. control 4.82 days).

**Fig. 2:**
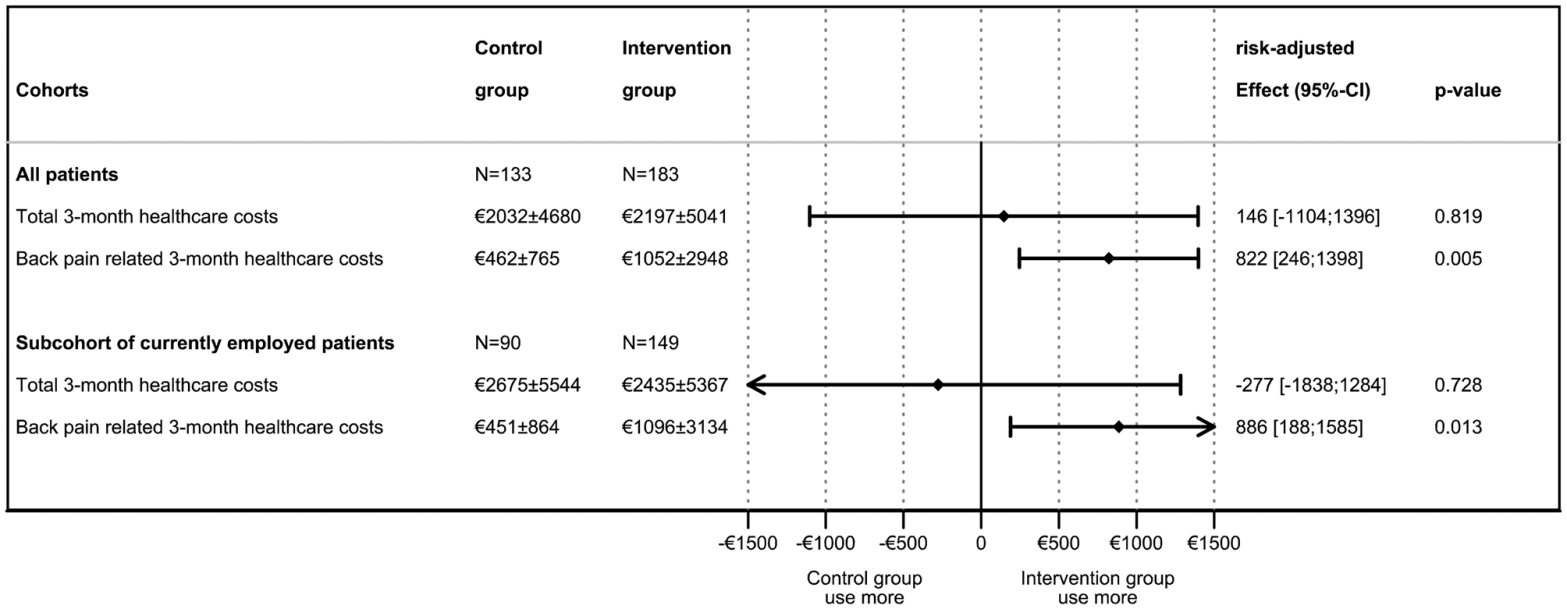
Adjusted differences in total and back pain-related costs

As shown in Fig. 2, the adjusted difference in total back pain-related costs, including the costs for general practitioner and specialist contacts, hospital and sick leave days, between the control and intervention groups amounted to €822.22 (*p* = 0.005, 95%CI 246.24;1398.20).

### Sensitivity analysis 1: Total healthcare service utilization in the overall cohort

Looking at the total healthcare service utilization, a different picture emerges with regard to sick leave days: While sick leave days are documented in the intervention group on average (5.38) in the 3-month period, these are comparable in the control group (4.82, see Fig. 1). The results of the statistical analysis adjusted for age and gender show only a slight increase in total costs: 145.88€ (*p* = 0.819, 95%CI -1104.10;1395.86).

### Sensitivity analysis 2: Sub-cohort of currently employed patients

Looking at the sub-cohort of currently employed patients, a similar picture emerges as in the main analysis: In the 3-month period, there are on average fewer contacts with general practitioners and specialists in the intervention group than in the control group. However, the average number of back pain-related sick days was again significantly higher in the intervention group than in the control group (see Fig. 1). The results of the statistical analysis confirm an intervention-related increase in back pain-related costs of €886.23 (*p* = 0.013, 95%CI 187.55;1584.91).

### Sensitivity analysis 3: Additional adjustment for prior healthcare utilisation

When the analysis was additionally adjusted for the total cost of healthcare utilisation in the three months prior to inclusion (Figure S1), the intervention-related increase in back pain-related costs remained statistically significant, amounting to €601 (95% CI 140;1061, *p* = 0.011) in the overall cohort and €677 (95% CI 36;1318, *p* = 0.039) in the sub-cohort of currently employed patients.

### Sensitivity analysis 4: Excluding F32.*/F33.*as back-pain proxies

In a further sensitivity analysis, removing the depressive-disorder codes (F32.*, F33.*) from the definition of back-pain-related care — while retaining the non-specific pain code R52.* — left the direction and the statistical significance of the cost difference essentially unchanged (full cohort: +€783, *p* = 0.009, vs. +€822, *p* = 0.005 in the main analysis). Details are reported in Supplementary Table S2.

### Cost-effectiveness analysis

As described above, an intervention-related increase in back pain-related service utilization of €822.22 (p = 0.005, 95%CI 246.24;1398.20) was identified in the main analysis. This contrasts with an intervention-related increase in ‘self-reported knowledge’ of 0.25 (p = 0.007; 95%CI 0.07;0.43) and an intervention-related increase in ‘perceived informedness’ of 0.51 (p = 0.009; 95%CI 0.13;0.89), as published in the main article of the study [18]. Combining these values results in additional costs per unit of the patients ‘self-reported knowledge” of €3328.54 (95%CI 903.08;13008.32) and additional costs per unit of patients ‘perceived informedness’ of €1611.21 (95%CI 434.27;6961.03).

## Discussion

**Fig. 3:**
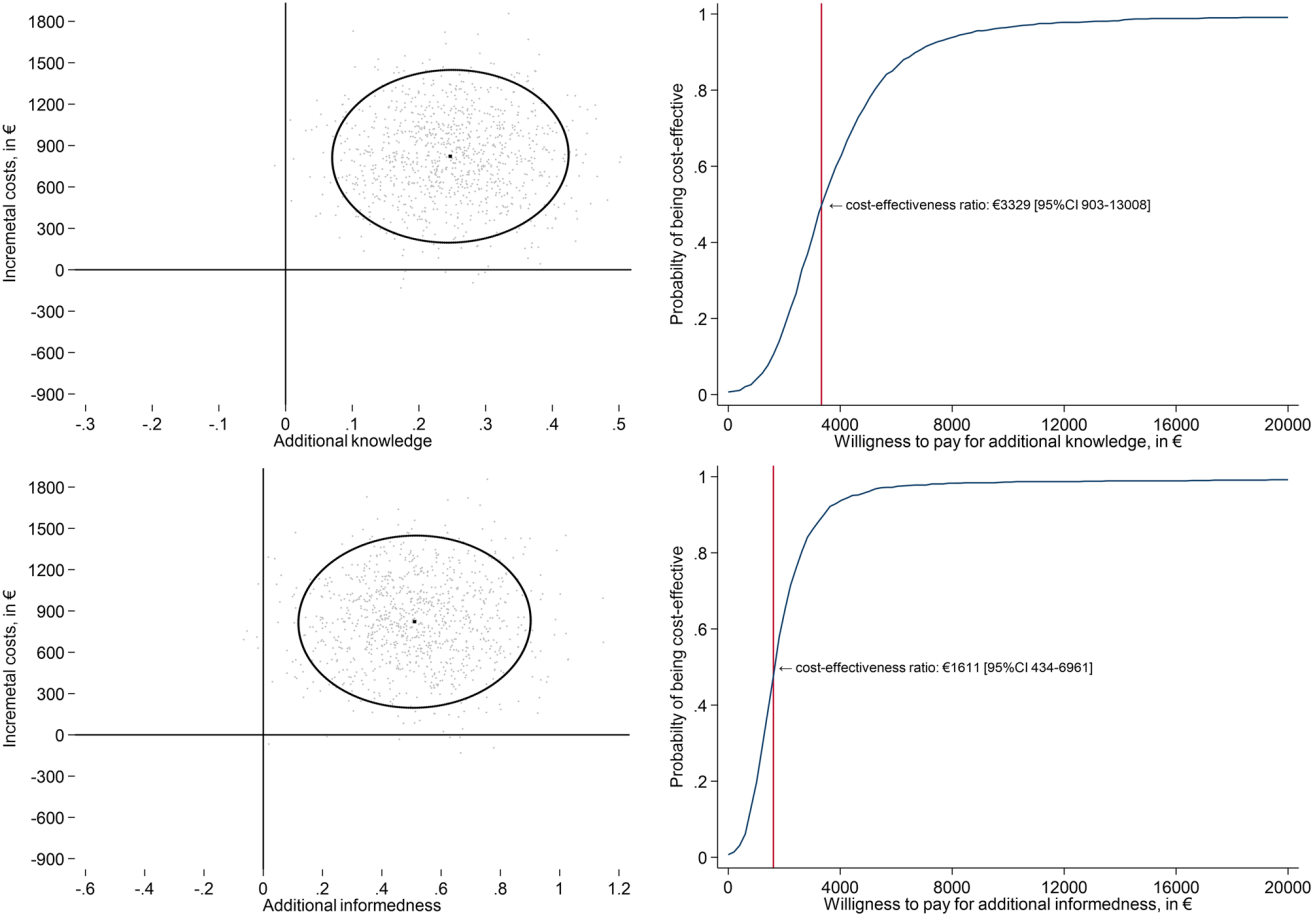
Cost-effectiveness planes and cost-effectiveness acceptability curves for ‘self-reported knowledge’ and ‘perceived informedness’

Because both effectiveness measures were developed de novo for this study and no willingness-to-pay thresholds exist for them, the cost-effectiveness analysis is exploratory and hypothesis-generating, and the resulting ratios cannot be judged against any benchmark of cost-effectiveness. They should be read as a descriptive juxtaposition of the incremental cost and the incremental effect on patients’ self-reported knowledge and perceived informedness, rather than as a verdict on value for money. The cost-effectiveness ratios mentioned above (€3329 for a one-unit increase in the patients ‘self-reported knowledge’ and €1611 for a one-unit increase in the patients ‘perceived informedness’) provide an initial insight into this, but leave open whether the mean value shown in these values is above or below the willingness-to-pay thresholds. In Fig. 3, this relationship is illustrated by plotting the probability that the intervention would be considered cost-effective across a range of hypothetical willingness-to-pay values. Because there are no established willingness-to-pay thresholds for increasing self-reported knowledge or perceived informedness among LBP patients in Germany, these curves are intended only to allow readers to locate the result against any threshold they consider relevant, not to demonstrate cost-effectiveness.

Overall, the provision of tala-med appears to be associated with higher back pain-related costs but with no increase in total healthcare costs. A key to interpreting this discrepancy lies in the contrast between back pain-related and total sick leave (Fig. 1). While back pain-coded sick leave was substantially higher in the intervention group (incidence rate ratio 8.73, *p* = 0.001), total all-cause sick leave was very similar between groups (5.38 vs. 4.82 days over three months), and total costs did not differ (€145.88, *p* = 0.819). Taken together, this pattern is more consistent with a reclassification of sick leave towards a back pain code (category substitution) than with a genuine increase in overall work absence; an eight-fold true increase in absence would be implausible for a brief educational intervention. A plausible mechanism is the documentation and coding behaviour incentivised by the German statutory system: physicians and patients in the intervention group, having engaged intensively with back pain through the study and the portal, may have been primed to record back pain as the certified primary reason for a sickness absence that would otherwise have been coded under a different or non-specific diagnosis. We therefore interpret the elevated back pain-related sick-leave figure primarily as a documentation effect rather than as evidence of a substantial increase in time off work, and we regard the stability of total costs as the more robust summary of the intervention’s short-term economic impact. We cannot, however, fully exclude a real component — for example, that increased awareness led some patients to take recommended periods of rest — and disentangling these explanations will require studies designed with sick-leave certification as a prespecified endpoint.

These positive effects on patients ‘perceived informedness’ align with findings from a qualitative interview study involving 32 patients from the intervention group [30]. Moreover, they are consistent with the outcomes of digital self-management programs, which have demonstrated benefits in enhancing understanding of LBP [31] and reducing pain intensity. Notably, a recent meta-analysis reports small but clinically meaningful improvements in pain intensity both immediately and shortly after such interventions [14]. Also in the GAP trial, a reduction of pain intensity was observed in the intervention group [27].

A major limitation of the study was that the primary outcome was assessed using newly developed scales for both indicators, which is susceptible to bias [32]. Both effectiveness measures used in the cost-effectiveness analysis were developed de novo for this study, which limits their construct validity and external comparability; the cost-effectiveness ratios are correspondingly exploratory. This was considered necessary due to the lack of suitable measures and cognitive pretests showed good internal consistency [18]. In addition, the observed reduction in pain severity and findings from the process evaluation [30] suggest that patients’ increased informedness was not merely subjective but led to behavioral changes that helped alleviate their LBP [18]. Second, another limitation is that our study only assessed the short-term effects of tala-med, given that the healthcare utilization costs were assessed for a three-month interval only. Relatedly, the study was not powered for the health economic outcomes. Given the high variance characteristic of cost data, the available sample of 183 intervention and 133 control patients may be underpowered to detect economically meaningful differences in cost, and the cost and cost-effectiveness results should be interpreted accordingly. Third, the analysis was restricted to routine claims data and the valuation of sickness absence. Costs outside this scope were not captured. In particular, additional physician time spent using the portal during the consultation, training and support activities, and the development, maintenance, and hosting costs of the portal were not quantified. A full implementation costing or societal-perspective economic evaluation would be required to capture these components. Fourth, from a methodological perspective, the cluster-randomised design resulted in an imbalance in the number of recruiting clusters contributing patients to the health-economic analysis. Although cluster-robust standard errors were used, residual cluster-level imbalance cannot be fully excluded. In addition, the cost analyses were restricted to complete cases with available claims data. This approach assumes that missingness was unrelated to the cost outcome; if this assumption does not hold, the estimates may be subject to bias. Reassuringly, the patients contributing to the health-economic analysis did not differ from the effectiveness-analysis population in age or sex, and the number analysed was essentially equal to the full recruited cohort, so that selective exclusion through unavailable claims data is unlikely to have materially biased the estimates. We cannot, however, exclude residual differences in characteristics not observed in both data sources, such as baseline LBP severity or employment status. Moreover, indirect costs were valued using the human capital method, which assumes full productivity loss for each day of certified absence and does not account for presenteeism, partial replacement, or compensation by colleagues. It therefore tends to overestimate indirect costs — a limitation of particular relevance here, as sick leave was the main driver of the back pain-related cost difference. Fifth, applicability is further restricted by the inclusion of patients insured with company health insurance funds (BKK) in Bavaria only. Because BKK members are predominantly in employment and may differ systematically from the general LBP population in occupational status, income, and healthcare access, the cost estimates — and in particular the sick-leave findings — may not generalise to other populations or insurance schemes. Sixth, adjustment for patient characteristics relied primarily on the cluster randomisation, supplemented by a propensity score for age and sex and, in a non-prespecified sensitivity analysis, by adjustment for prior healthcare utilisation. Residual within-practice confounding by characteristics not captured by these variables — for example pain chronicity, comorbidity, or employment sector — cannot be fully excluded. Seventh, the definition of back pain-related utilisation involved a prespecified but partly discretionary choice of diagnosis and procedure codes. In particular, the inclusion of depressive (F32, F33) and non-specific pain (R52) codes may have captured some utilisation that is not exclusively attributable to back pain. Because a large number of alternative code definitions would be defensible, we did not privilege any single exclusion analysis, but we acknowledge that the cost estimates are sensitive to these definitional choices. To address this directly for the conceptually most distinct codes, we conducted a targeted sensitivity analysis excluding the depressive-disorder codes (F32.*, F33.*) while retaining R52.*; this changed the back-pain cost estimate only marginally and did not alter its direction or significance (Supplementary Table S2). Finally, our study design does not allow us to differentiate the contributions of the two intervention components: the use of the portal during consultations and its subsequent use by patients at home.

## Supplementary Information

Below is the link to the electronic supplementary material.

Supplementary Material 1

## Data Availability

The datasets used and/or analysed during the current study available from the corresponding author on reasonable request.
