# Supplementary material for: The economic impact of a physician-introduced web portal on lower back pain: results from a cluster-randomized trial
Source: Cost Eff Resour Alloc. 2026 Jul 7;24:84. doi: 10.1186/s12962-026-00796-z (PMC13343678; doi:10.1186/s12962-026-00796-z)
Supplement: Supplementary file 1 — Supplementary Material 1 [file 12962_2026_796_MOESM1_ESM.pdf]

Table S1: The identification of back pain-related costs for the healthcare system was based on the following predefined main diagnoses and procedures:

Main diagnoses (ICD-10)

- M40-M54 Diseases of the spine and back
- F33.* Recurrent depressive disorder
- F32.* Depressive episode
- R52.* Pain, not elsewhere classified

Procedures (OPS)

- 5-83* Operations on the spine
- 3-203* Native computed tomography of the spine and spinal cord
- 3-223* Computed tomography of the spine and spinal cord with contrast medium
- 1-404.0 Percutaneous (needle) biopsy of intraspinal tissue: spinal cord
- 1-404.1 Percutaneous (needle) biopsy of intraspinal tissue: Spinal cord nerves and dorsal root ganglia, intraspinal
- 1-404.2 Percutaneous (needle) biopsy of intraspinal tissue: spinal meninges
- 1-480.4 Percutaneous (needle) biopsy of bone: Spine
- 1-481.4 Biopsy without incision of bone with guidance by imaging techniques: Spine
- 1-483.5 Percutaneous (needle) biopsy of joints and bursae: Joint of the spine
- 1-484.5 Percutaneous biopsy of joints and bursae with imaging guidance: Joint of the spine
- 1-512.0 Percutaneous biopsy: Spinal cord
- 1-512.1 Percutaneous biopsy: Intraspinal parts of spinal cord nerves and spinal ganglia
- 1-512.2 Percutaneous biopsy: Spinal cord membranes
- 1-512.3 Percutaneous biopsy: Diagnostic opening of the spinal canal
- 1-503.4 Biopsy of bone: Spinal column
- 1-504.5 Bone biopsy: Joint of the spine
- 1-697.5 Diagnostic arthroscopy: Joint of the spine
- 1-799 Instrumental 3D functional analysis of the spine
- 1-854 Diagnostic percutaneous puncture: joint in the spine

The identification of days of incapacity for work due to back pain was based on the following predefined outpatient diagnoses (ICD-10):

- M450-M54 Diseases of the spine and back
- F33.* Recurrent depressive disorder
- F32.* Depressive episode
- R52.* Pain, not elsewhere classified
- F45.4- Persistent pain disorder
- F45.40 Persistent somatoform pain disorder
- F45.41 Chronic pain disorder with somatic and psychological factors
- Z56 Contact causes related to working life (Note: corresponds to burn out, rarely coded)

Figure S 1: Adjusted differences in total and back pain-related costs (adjusted also for the total costs of healthcare utilization prior to the inclusion in the study)

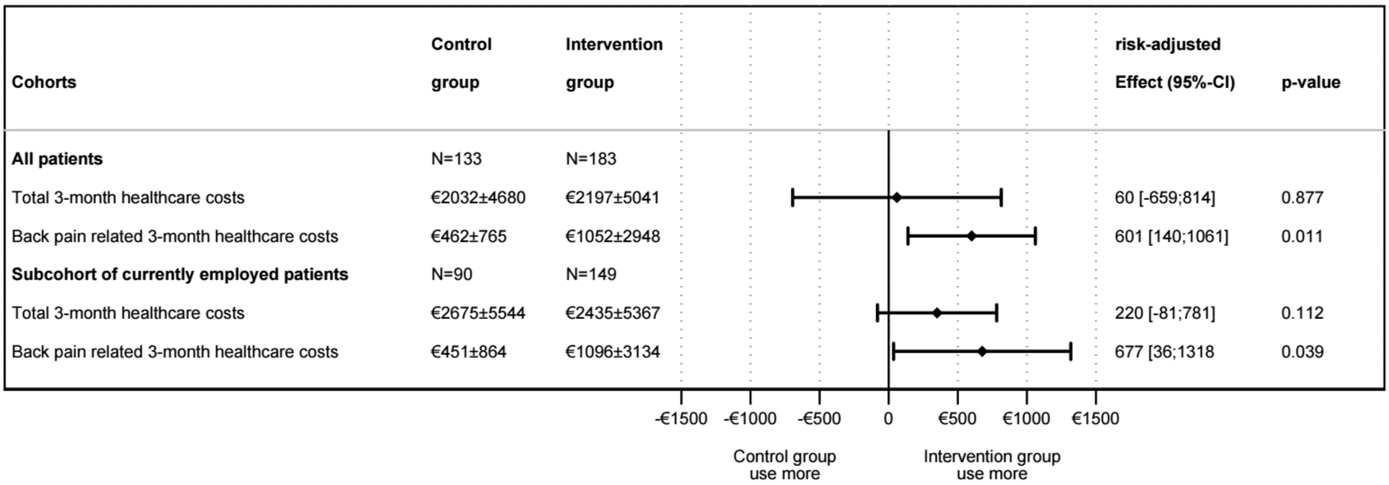

CI = confidence interval. Estimates are average marginal effects of the intervention on total and back-pain-related costs from two-part models (logit + gamma/log GLM), adjusted for the propensity score and 3-month healthcare utilization prior to the inclusion in the study, with standard errors clustered at the physician level. A positive value indicates higher costs in the intervention group.

Table S2: Risk-adjusted difference in back-pain-related costs — main analysis vs. sensitivity analysis excluding F32.*/F33.*

Cohort	Analysis	Risk-adjusted incremental cost, € (95% CI)	p-value
All patients (N = 316)	Main (incl. F32.*/F33.*)	+822 (246 to 1398)	0.005
All patients (N = 316)	Sensitivity (excl. F32.*/F33.*)	+783 (198 to 1368)	0.009
Currently employed subcohort (N = 239)	Main (incl. F32.*/F33.*)	+886 (188 to 1585)[1]	0.013
Currently employed subcohort (N = 239)	Sensitivity (excl. F32.*/F33.*)	+830 (120 to 1540)[1]	0.022

CI = confidence interval. Estimates are average marginal effects of the intervention on back-pain-related costs from two-part models (logit + gamma/log GLM), adjusted for the propensity score, with standard errors clustered at the physician level. A positive value indicates higher costs in the intervention group.

Note: The back-pain-related cost endpoint reported in the main analysis includes a small number of diagnostic codes that are not exclusively musculoskeletal, namely the depressive-episode codes (F32.*), the recurrent-depressive-disorder codes (F33.*) and the non-specific pain code (R52.*). To assess whether the inclusion of the depression codes materially influenced the back-pain cost estimate, we conducted a sensitivity analysis in which all F32.* and F33.* codes were removed from the definition of back-pain-related care, while R52.* and all musculoskeletal (M..) and back-related DRG codes were retained. The depression codes contribute to the back-pain cost endpoint through two components: inpatient costs (hospital cases carrying a back-pain diagnosis) and back-pain-related sick-leave days (valued at €332 per day). For the sensitivity endpoint we removed, at the patient level, the inpatient costs and the sick-leave days that were attributable solely to an F32.*/F33.* code — i.e., hospital cases and sick-leave spells that no longer qualified as back-pain related once the depression codes were excluded. Cases and spells that also carried a musculoskeletal code were retained unchanged. All remaining cost components (general-practitioner and specialist contacts) are not back-pain specific and were therefore unaffected. The two-part models (logit first part; gamma GLM with log link, second part), the propensity-score adjustment and the clustering of standard errors at the physician level were identical to the main analysis. The depression codes contributed only marginally to the back-pain cost endpoint (mean €3.67 of inpatient cost and 0.08 sick-leave days per patient).
